# Short term visual and structural outcomes of anti-vascular endothelial growth factor (anti-VEGF) treatment delay during the first COVID-19 wave: A pilot study

**DOI:** 10.1371/journal.pone.0247161

**Published:** 2021-02-17

**Authors:** Ameay V. Naravane, Rusdeep Mundae, Yujia Zhou, Christopher Santilli, Frederik J. G. M. van Kuijk, Hossein Nazari, Justin Yamanuha, Geoffrey G. Emerson, Dara D. Koozekanani, Sandra R. Montezuma

**Affiliations:** 1 Department of Ophthalmology and Visual Neuroscience, University of Minnesota, Minneapolis, Minnesota, United States of America; 2 University of Minnesota Medical School, Minneapolis, Minnesota, United States of America; 3 The Retina Center, Minneapolis, Minnesota, United States of America; Massachusetts Eye & Ear Infirmary, Harvard Medical School, UNITED STATES

## Abstract

Regularly scheduled intravitreal anti-vascular endothelial growth factor (anti-VEGF) injections are essential to maintaining and/or improving many ocular conditions including: neovascular age-related macular degeneration (nAMD), diabetic retinopathy, and retinal vein occlusions with macular edema (RVO). This study aims to assess the effect of unintended delays in anti-VEGF treatment during the first wave of the COVID-19 pandemic. This retrospective case series identified patients receiving regularly scheduled anti-VEGF intravitreal injections based on current procedural terminology (CPT) code at two practices in Minnesota. Diagnoses were limited to nAMD, diabetic macular edema (DME), proliferative diabetic retinopathy, and RVO. Patients were divided into two groups based on whether they maintained or delayed their follow-up visit by more than two weeks beyond the recommended treatment interval during the COVID-19 lockdown. The ‘COVID-19 lockdown’ was defined as the period after March, 28^th^, 2020, when a lockdown was declared in Minnesota. We then compared the visual acuity and structural changes to the retina using ocular coherence tomography (OCT) to assess whether delayed treatment resulted in worse visual outcomes. A total of 167 eyes from 117 patients met criteria for inclusion in this study. In the delayed group, the average BCVA at the pre- and post-lockdown visits were 0.614 and 0.715 (logMAR) respectively (p = 0.007). Central subfield thickness (CST) increased from 341 to 447 in the DME delayed group (p = 0.03) while the CST increased from 301 to 314 (p = 0.4) in the nAMD delayed group. The results of this pilot study suggests that treatment delays may have a negative impact on the visual and anatomic outcomes of patients with nAMD and DME. Future studies with larger sample sizes are required for further investigation.

## Introduction

As of December 4^th^, 2020 there were 14 million confirmed cases and 275,000 deaths due to the novel Coronavirus Disease 19 (COVID-19) in the United States [1]. In Minnesota, a total of 338,000 cases and 3,800 deaths were reported during the same time period [2]. In response to the COVID-19 pandemic, on March 27th, 2020, the American Academy of Ophthalmology (AAO) published a list of emergent and urgent procedures performed in an operating room or ambulatory center that should not be postponed. Intravitreal anti-vascular endothelial growth factor (anti-VEGF) injections were not included on this list [3]. With the exception of these guidelines, there was limited recommendations on the management of intravitreal injections during the COVID-19 pandemic [4].

Many clinics continued to perform intravitreal injection with each developing its own protocols for anti-VEGF office procedures balancing vision-saving therapy with the risk of COVID-19 exposure [5, 6]. Management strategies aimed to minimize risk of exposure to COVID for both the patients and healthcare workers [7]. For example, the American Society of Retina Specialists (ASRS) Preferences and Trends (PAT) survey [8] showed that prior to the pandemic, most retinal specialists conducted an ocular exam and performed an OCT at every visit. During the pandemic, retina specialists modified clinical practice to either order fewer OCT images or plan for injection only visits. If an OCT was required for a treatment decision, appropriate measures were taken to ensure the safety of patients and clinic staff (e.g. use of masks, cleaning of machines). Globally, some retinal specialists even modified protocol to only perform intravitreal injections in the operating room (OR) on one eye at a time [6, 8].

Despite many clinics’ efforts to provide a safe environment for intravitreal injections during the pandemic, patient adherence to treatment regimens declined during the initial wave of the COVID-19 pandemic. By comparing the total number of intravitreal injection visits from March 15^th^ to April 14^th^ in 2020 to the same time period over the past four years, Wasser et al estimated a 50% reduction in the number of clinic visits for intravitreal injections in 2020 [9].

One possible explanation for this drop in patient adherence is fear of contracting COVID-19. Patients over the age of 65 comprise the majority of the 2 million intravitreal anti-VEGF injections performed annually in the United States [10–12]. This group has been found to be 90 times more likely to succumb to COVID-19 compared to those aged 18–29 years [13]. As a result, this patient population may have delayed treatment for conditions such as diabetic macular edema (DME), neovascular AMD (nAMD), and retinal vein occlusions (RVO) during the COVID-19 pandemic, resulting in potential vision loss [11, 12].

This study compares the visual and structural (OCT) data in individuals with no lapse in post-lockdown anti-VEGF treatment to those who delayed their post-lockdown follow-up by greater than two weeks. We defined the “COVID lockdown” era as after March 28th, 2020, in Minnesota [14]. The goal of this study was to report outcomes of delayed intravitreal anti-VEGF injection intervals for patients with exudative macular diseases (neovascular AMD, DME, RVO) or proliferative diabetic retinopathy (PDR).

## Methods

The protocol was approved by the Institutional Review Board (IRB) of the University of Minnesota and adhered to the Health Insurance Portability and Accountability Act as well as the tenets of the Declaration of Helsinki. The IRB issued a waiver of the requirement for consent. A retrospective review of all patients who had follow up appointments with the retinal specialists of this study for intravitreal injection scheduled after March 28^th^, 2020 at the University of Minnesota Retina Clinic and the Retina Center in Minneapolis was performed by querying the electronic medical record (EMR) for the CPT code 67028 (intravitreal injection). The “pre-lockdown” period was defined as December 1^st^, 2019 through March 26^th^, 2020. We anticipated this patient cohort to have follow up scheduled during the early months of the COVID pandemic. The “post-lockdown” period was defined as March 28^th^, 2020 through September 30^th^, 2020.

The “pre-lockdown” visit was defined as the most recent appointment prior to the start of the lockdown period during which an ocular coherence tomography (OCT) was obtained. The “post-lockdown” visit was defined as the first visit immediately after the declaration of the lockdown period during which an OCT was obtained. Patients were divided into two groups: control and delayed. The control group was defined as those patients who did not miss/delay their follow-up visit by greater than two weeks past the recommended treatment interval (i.e. the recommended treatment interval was the same time frame in weeks recommended between the “pre-lockdown” visit and the follow-up visit. The delayed group was defined as those patients who delayed or rescheduled their follow-up visit by greater than two weeks beyond the recommended treatment interval.

Patients were excluded if they were less than 18 years old, had not been receiving intravitreal anti-VEGF injections for at least one year, or if their primary diagnosis was not nAMD, DME with or without PDR, or RVO. Patients were also excluded if they opted out of having their records used for research. Patients were followed by the retinal specialists involved in this study and were primarily on treat and extend regimens. The preferred anti-VEGF agents used at this medical center are bevacizumab (Avastin ®) and aflibercept (Eylea ®).

Baseline demographic data collected included age at pre-lockdown visit, gender, race, eye(s) involved, treating diagnosis, anti-VEGF agent, mean systolic and diastolic blood pressure, Hemoglobin A1c (%), lens status, and intraocular pressure (IOP) at pre- and post-lockdown visits. The recommended treatment interval (i.e. follow up interval) and best corrected visual acuity (BCVA) at the pre-lockdown and follow-up visits were reported along with the number of weeks that the follow-up visit was delayed (if applicable). OCT data collected included the CST) at the pre-lockdown and follow-up appointments. Among patients with DME, a greater than 10% change in CST was considered significant per DRCR.net guidelines for clinical significance [15].

Mean and range were reported for continuous variables. BCVA was converted to a logMAR equivalent for statistical analysis. A paired t-test was used to determine the statistical significance of continuous variables. McNemar’s Chi-Square Test was used to determine the statistical significance of paired categorical variables. Statistical analyses were performed using Microsoft Office 365 Excel © and plotted using GraphPad Prism 9. A significance level of 0.05 was chosen for all analyses.

## Results

A total of 167 eyes from 117 patients met criteria for inclusion in this study. This included 90 eyes from 60 patients in the control group and 77 eyes from 57 patients in the delayed group. The average age in the control group was 72 years (31–97 years) and 73 years (29–97 years) in the delayed group. There was no significant difference in the demographic characteristics between the control group and delayed group (Table 1). The last anti-VEGF agent used prior to the pre-lockdown visit was: aflibercept (45% of patients), bevacuzimab (43%), ranibizumab (Luncentis ®) (7%), and brolucizumab (Beovu ®) (5%).

**Table 1:** Demographic characteristics of study population.

	Control Group %, (n =)	Delayed Group %, (n =)	Significance
**Overall**			
Total patients	60	57	
Total eyes	90	77	
Age (range)	72 (31–97)	73 (29–97)	p = 0.4
Female	45% (n = 27)	61% (n = 35)	p = 0.07
A1c (Avg)	6.8% (n = 37)	7.2% (n = 36)	
Blood Pressure (Avg)	135/74 (n = 53)	136/75 (n = 53)	
**Ophthalmic**			
OD	49% (n = 44)	42% (n = 32)	
Phakic	32% (n = 29)	35% (n = 27)	
IOP pre-lockdown	14	16	
IOP follow-up	15	15	
**Treating Diagnosis**			
nAMD	57	36	
DME +/- PDR	30	34	
RVO	3	7	

There was no significant difference in the demographic characteristics between the control group and the delayed group. OD: right eye, DME: diabetic macular edema, PDR: proliferative diabetic retinopathy

The BCVA was calculated at the pre-lockdown and follow-up visits among the delayed and control groups. In the control group, the average BCVA at the pre-lockdown and follow-up visits were 0.404 and 0.413 (logMAR) respectfully (p = 0.70). In the delayed group, the average BCVA at the pre-lockdown and follow-up visits were 0.631 and 0.767 (logMAR), respectively (p = 0.007). Subgroup analysis was also performed for both groups by treating diagnosis and summarized in Table 2.

**Table 2:** BCVA (logMAR) at the pre-lockdown and follow-up visits among the control and delayed groups.

	Pre-Lockdown BCVA (logMAR)	Follow up BCVA (logMAR)	Significance
**Control Group (n = 90)**	**0.404**	**0.413**	**p = 0.70**
nAMD (n = 57)	0.391	0.419	p = 0.20
DME (n = 30)	0.417	0.449	p = 0.40
RVO (n = 3)	0.333	0.272	Not significant
**Delayed Group (n = 77)**	**0.631**	**0.767**	**p = 0.007**
nAMD (n = 36)	0.729	0.853	p = 0.04
DME (n = 34)	0.544	0.722	p = 0.06
RVO (n = 7)	0.555	0.549	Not significant

There was no significant difference between the BCVA at the pre-lockdown (**0.404**) and follow-up (**0.413**) in the control group **(p = 0.70).** BCVA worsened in the delayed group from **0.631** (pre-lockdown) to **0.767** (follow-up). This was statistically significant **(p = 0.007).** Subgroup analysis was also performed for nAMD and diabetes in both groups and there was a trend towards worsening BCVA in the delayed group.

**Fig 1:**
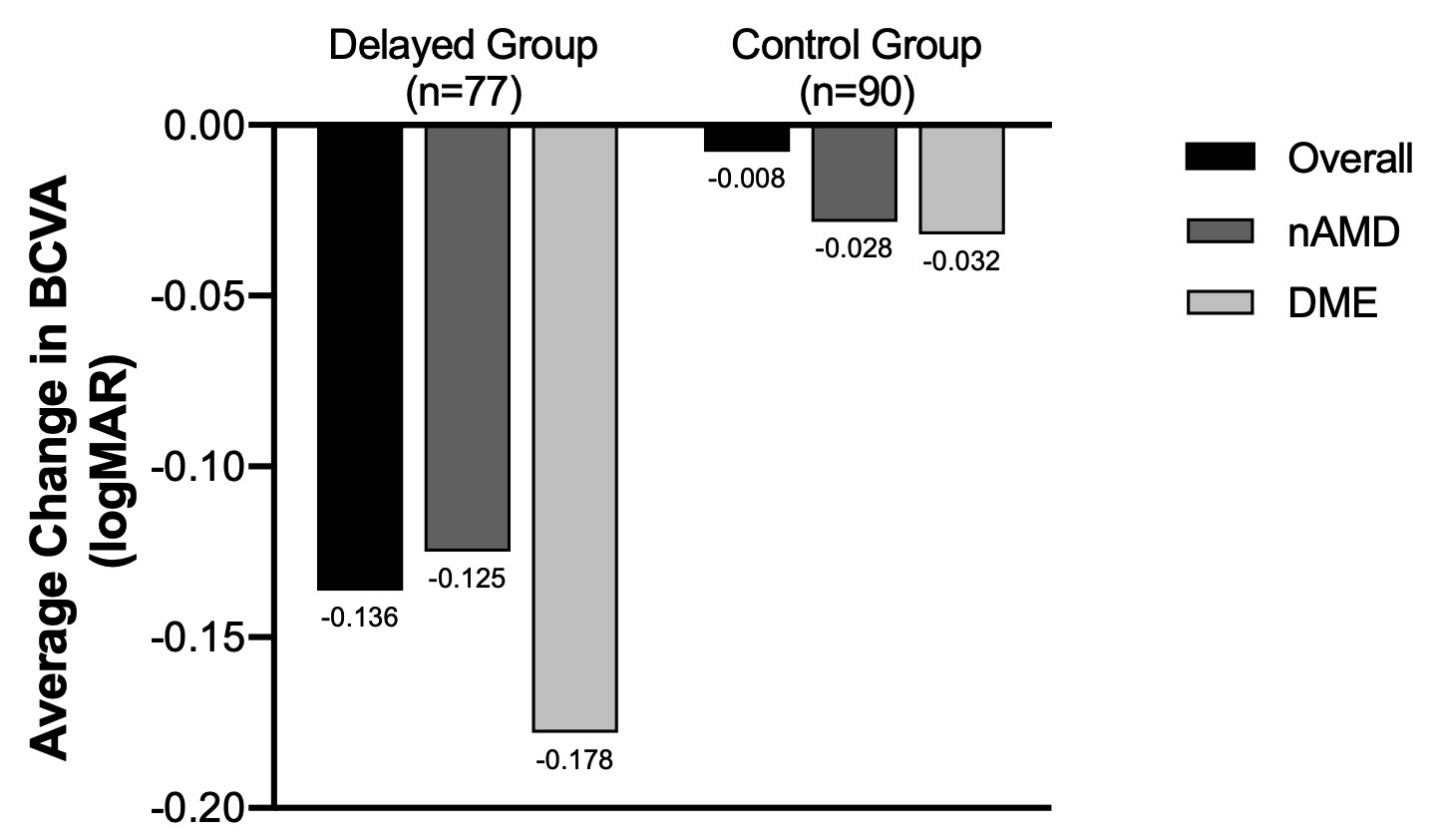
Delta BCVA (logMAR) (pre-COVID BCVA–follow-up BCVA) in the control and delayed groups. BCVA worsened in the **overall delayed group** by an average of 0.136 and by an average of 0.008 in the **overall control group (p = 0.02)**. This corresponds to a difference of approximately one line (or 5 letters). BCVA worsened in the **delayed group with nAMD** by an average of 0.125 and by an average of 0.028 in **the control group with nAMD** (p = 0.07). BCVA worsened in the **delayed group with DME** by an average of 0.178 and by an average of 0.032 in **the control group with DME** (p = 0.162).

The average change in BCVA was measured for both the control and delayed groups between the pre-lockdown and follow-up visits. BCVA (logMAR) worsened by an average of 0.008 in the control group and by 0.136 in the delayed group (p = 0.02). This corresponds to a difference of approximately one line (or 5 letters) between the control group and delayed group. Subgroup analysis for patients with nAMD and DME was also performed. Among patients with nAMD, BCVA worsened in the delayed group by an average of 0.125 and by an average of 0.028 in the control group (p = 0.07). Among patients with DME, BCVA worsened in the delayed group by 0.178 and worsened by 0.032 in the control group (p = 0.16). These data are summarized in Fig 1.

The recommended treatment interval after the pre-lockdown and follow up visits were identified for the delayed and control groups. The change in the recommended treatment interval from the pre-lockdown to follow up visit was recorded. In the control group, the recommended treatment interval decreased by 0.2 weeks (p = 0.4) and decreased by 0.3 weeks in the delayed group (p = 0.6).

**Fig 2:**
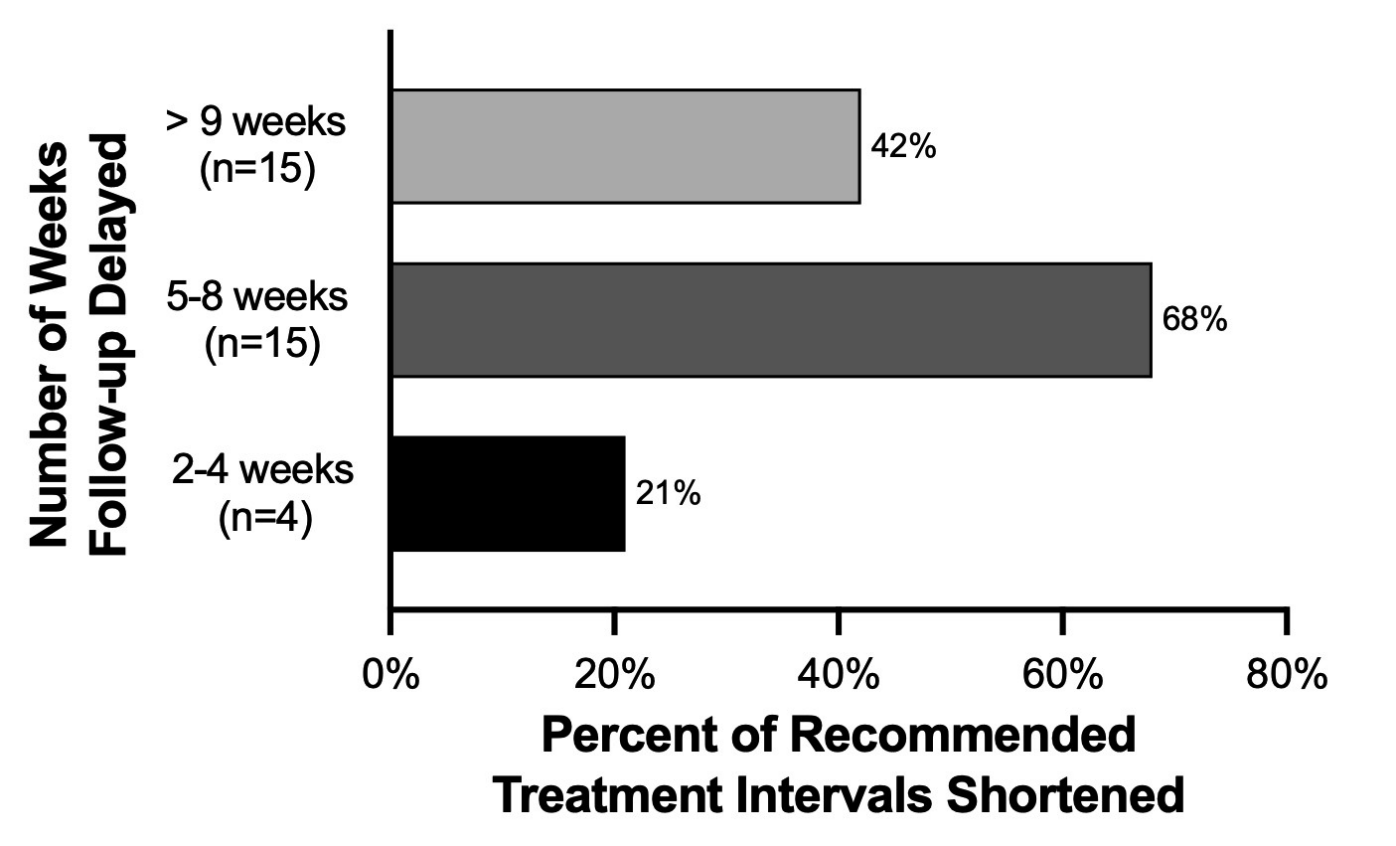
Percent of treatment intervals shortened stratified by number of weeks follow-up was delayed.

The delayed group was further divided into three subgroups based on the number of weeks their follow-up visit was delayed: 2–4 weeks (n = 19), 5–8 weeks (n = 22), or greater than 9 weeks (n = 36). The percentage of recommended treatment intervals that were shortened for these groups was also analyzed. In the group delayed by 2–4 weeks, 21% (n = 4) of treatment intervals shortened by an average of 4.5 weeks. In the group delayed by 5–8 weeks, 68% (n = 15) were shortened by an average of 3.1 weeks. Finally, in the group delayed by greater than 9 weeks, 42% (n = 15) were shortened by 3.1 weeks. These results are summarized in Fig 2.

**Fig 3:**
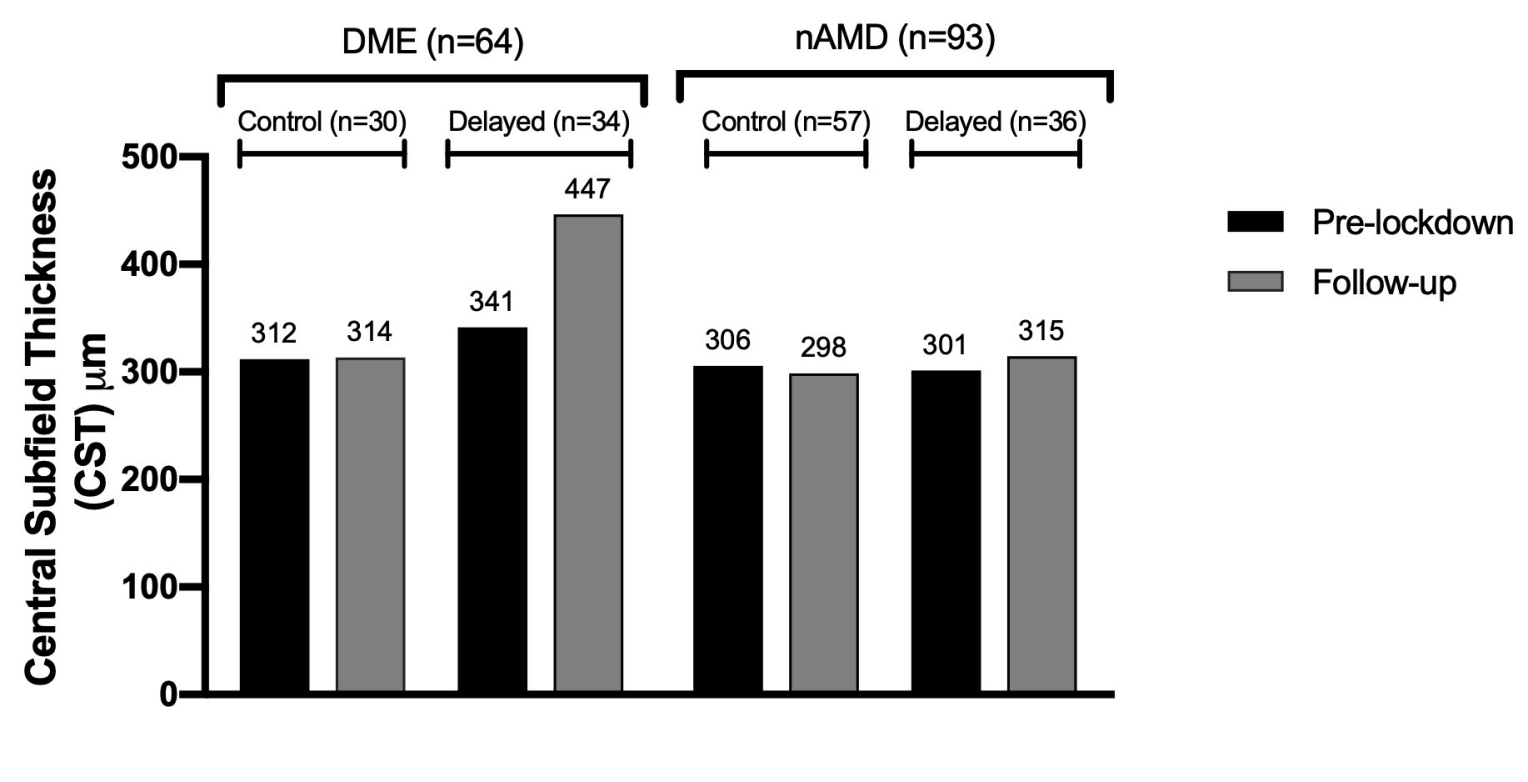
OCT analysis of patients with DME and nAMD at pre- and follow-up visits among control and delayed groups. The most recent OCTs completed pre-lockdown was compared to the OCT at next subsequent follow-up. Central subfield thickness (CST) was measured. For patients with DME, a change in CST greater than 10% was noted per DRCR.net criteria for clinical significance. **Among patients with DME,** CST changed from 311 (pre-lockdown) to 314 (follow-up) (p = 0.9) in the control group and from 341 (pre-lockdown) to 447 (follow-up) (p = 0.03) in the delayed group. **Among patients with nAMD,** CST changed from 305 (pre-lockdown) to 298 (follow-up) in the control group (p = 0.4), and from 301 (pre-lockdown) to 314 (follow-up) in the delayed group (p = 0.4).

**Fig 4:**
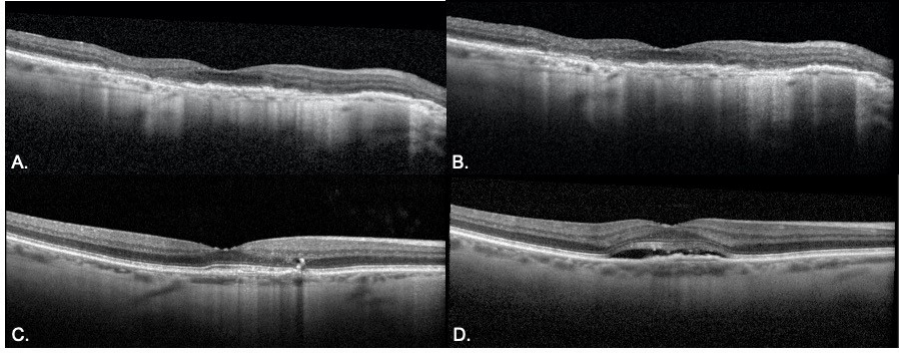
Example of nAMD disease progression on OCT from pre-lockdow to follow-up visit in the delayed and non-delayed groups. **A-B.** A representative patient with nAMD presented pre-lockdown visit (A) with controlled nAMD. The patient returned for a 6 week follow up with stable disease (B). **C-D.** Another patient with nAMD presented for pre-lockdown visit with stable disease (C) with a plan for follow-up in 4 weeks. Follow-up was delayed by 6 weeks and OCT revealed significant accumulation of subretinal fluid.

**Fig 5:**
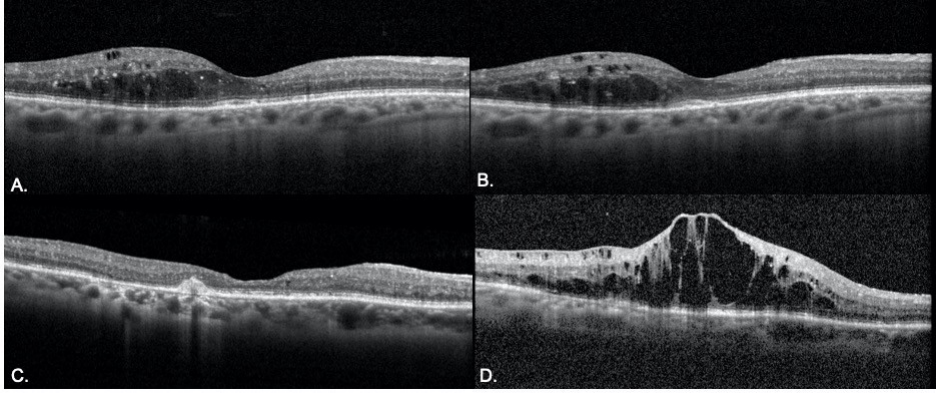
Example of DME disease progression on OCT from pre- to follow-up visit in the delayed and non-delayed groups. **A-B**. A patient with DME presented (A) for pre-lockdown visit with stable disease. The patient returned for 10 week follow-up (B) with a stable degree of intraretinal fluid. **C-D.** Another patient presented for a pre-lockdown visit with stable disease (C) with a plan for follow up in 8 weeks. Follow up was delayed by 6 weeks and OCT revealed significant accumulation of intraretinal and subretinal fluid.

Finally, OCTs were analyzed by primary diagnosis for both the control and delayed groups. For patients with DME, the central subfield thickness (CST) increased from 311 to 314 microns (p = 0.9) in the control group and increased from 341 to 447 microns (p = 0.03) in the delayed group. In the control group, CST increased by greater than 10% in 13% (n = 4) of OCTs. In the delayed group, CST was worsened by greater than 10% in 35% (n = 12) of OCTs. For patients with nAMD, the CST decreased from 305 to 298 microns (p = 0.4) in the control group and increased from 301 to 314 microns (p = 0.4) in the delayed group. Additional characteristics from OCT analysis are described in Fig 3. OCT findings demonstrating the impact of treatment delay for patients with nAMD and DME are shown in Figs 4 and 5, respectfully.

## Discussion

The optimal treatment regimen for neovascular AMD and DME remains debated. However, there has been a trend towards maximizing clinical benefit while reducing treatment burden (treat and extend). The two landmark studies of patients with exudative age-related macular degeneration (nAMD), MARINA [16] and ANCHOR [17], demonstrated that monthly injections with ranibizumab maintained visual acuity in 90% of patients at two years. Subsequent large cohort studies which compared ranibizumab with off-label bevacizumab in the United States (CATT) [18] and the United Kingdom (IVAN) [19] showed that less frequent dosing with either medication can maintain similar visual acuity as monthly dosing at one year.

Similarly, the RISE and RIDE clinical trials initially demonstrated efficacy of monthly ranibizumab injections for DME [20]. The VIVID and VISTA trials subsequently showed that aflibercept given every 4 weeks and every 8 weeks had similar efficacy in visual improvement of DME patients at 148 weeks [21]. A multicenter randomized study of ranibizumab for DME further demonstrated that a treat-and-extend protocol can achieve the same gain of BCVA with a significantly lower number of injections at 1 year [22]. However, even with the treat and extend regimen, regimens must be lengthened in a controlled manner with close follow-up. Most retina specialists adjust treatment intervals by one or two weeks at a time for fear of irreversible visual consequences with longer extensions.

To the authors’ knowledge, this is the first study to evaluate both the short term visual and anatomic impact of delays in intravitreal anti-VEGF injections during the COVID-19 pandemic. Rahimzadeh et al evaluated the impact of delayed treatment on BCVA among patients with newly diagnosed DME and nAMD during the COVID-19 pandemic and found no significant change [23]. In this retrospective pilot study, we found that patients whose appointments were delayed by greater than two weeks due to the COVID-19 pandemic had worse visual outcomes compared to those who attended their scheduled appointments. Further subset analysis revealed this was significant for patients with macular degeneration (p = 0.04) and was not significant for patients with diabetic macular edema (p = 0.06). Regarding anatomical outcomes, we found that diabetic patients in the delayed group suffered an increase in CST compared to the control group. There was no statistically significant change observed in CST in the nAMD group in those who attended visits as recommended or delayed their return visits.

The literature regarding the impact of delayed anti-VEGF treatment on visual and anatomical outcomes in patients with DME is limited. Yalamanchili et al. found no statistically significant difference in BCVA between patients who unintentionally delayed treatment by at least 3 months and those who maintained their regular appointments [24]. When CST was stratified by ‘clinical significance’ (defined as a greater than 20 μm change in CST), the study demonstrated a statistically significant change: 24% of the non-delayed group had a clinically significant change in CST compared to 50% of the delayed group. These anatomical outcomes of Yalamanchili et al. are difficult to interpret in the context of our study due to the more liberal definitions of ‘treatment delay’ and ‘clinically significant’ change in CST [14]. Although Yalamanchili did not note a significant worsening in BCVA, the results of our study trended towards a statistically significant worsening in BCVA. In addition, Weiss et al also found a significant negative correlation between the number of breakoff periods (defined as a greater than 3 month delay) and change in visual acuity.

Among patients with nAMD with a treatment lapse of greater than 3 months, macular thickness tends to normalize after resuming anti-VEGF therapy while BCVA worsens by one full line of vision [25]. While the OCT analysis from our study did not show a significant change in CST in the delayed group, Greenlee et al demonstrated that patients who delayed treatment experienced an increase in CST in comparison to the control group. The likely reason for this difference is the shorter minimum delayed period used in our study (two weeks) compared to that used by Greenlee et al. Long term clinical follow up was not possible for our pilot study given the time period selected.

Using data from CATT, Ramakrishnan et al found that nAMD patients who missed their appointments by more than 36 days suffered worse visual outcomes compared to those who were on time [26]. The study is limited by relatively low missed appointments (10%). In addition, missed visits were attributed to a variety of factors and possibly confounding variables including age, grade of visual impairment, and increased distance between the residence and clinic. Nonetheless, these results are in line with the findings presented herein and suggest that treatment delay is associated with worsening VA.

Lastly, we observed a trend between change in recommended treatment intervals and duration of delay in visits. For patients whose visits were delayed by greater than four weeks, a larger portion had intervals shortened compared to those who attended visits as scheduled. This suggests that treatment delay greater than 4 weeks may result in worsened visual acuity and necessitate a change in treatment regimen by the provider in the short term. The data from this study will provide patients and providers better insight into the risks and benefits of missing treatment when faced with barriers to care, whether that barrier be transportation, insurance, or a global pandemic.

This study had several limitations. First, the data for the study was from multiple different providers at two different vitreoretinal clinics in Minnesota. The multiple providers in the study may have different thresholds for modifying treatment intervals. The study also had a small sample size of a relatively homogenous population. Given the time period of the study, (March 2020—September 2020), another limitation was the lack of data regarding the long term consequences of treatment delay. Data from subsequent follow up visits after the initial treatment delay were not collected. We also recognize that while some of the comparisons may have reached statistical significance when comparing delayed and control groups, these may not have long term clinical relevance. Finally, although patients in this study had similar demographic characteristics, patients from the two arms of this study were not matched. In addition, although a larger clinical study from a national database like the American Academy of Ophthalmology IRIS® Registry (Intelligent Research in Sight) or from the Komodo health data could provide meaningful statistical analysis, such study would have other limitations in terms of imaging, visual acuity and medical records access, giving our study a unique strength.

Despite recent onset of vaccine distribution, the COVID-19 pandemic will likely continue to impact patients with retinal diseases requiring regularly scheduled anti-VEGF treatment for at least the next year to come. The results of this pilot study show that delaying anti-VEGF treatment may have a negative impact on the visual and anatomic outcomes of patients with retinal disease in the short term. Future studies with a larger sample size are required to make more definitive conclusions.

## Supporting information

S1 Fig (PNG)
