# Supplementary figures and images for: Short term visual and structural outcomes of anti-vascular endothelial growth factor (anti-VEGF) treatment delay during the first COVID-19 wave: A pilot study
Source: PLoS One. 2021 Feb 17;16(2):e0247161. doi: 10.1371/journal.pone.0247161 (PMC7888661; doi:10.1371/journal.pone.0247161)

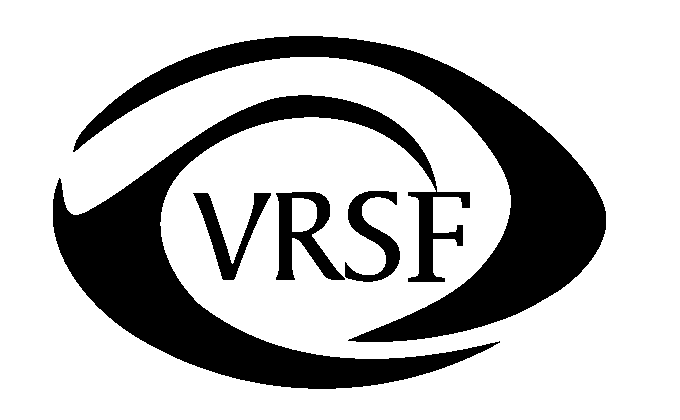

Supplement: S1 Fig — (PNG) [file pone.0247161.s001.png]
